# Curcumin Inhibits Lysophosphatidic Acid Mediated MCP-1 Expression via Blocking ROCK Signalling

**DOI:** 10.3390/molecules26082320

**Published:** 2021-04-16

**Authors:** Ying Zhou, Peter J. Little, Suowen Xu, Danielle Kamato

**Affiliations:** 1 School of Pharmacy, Pharmacy Australia Centre of Excellence, The University of Queensland, Woolloongabba, QLD 4102, Australia; ying.zhou@uq.edu.au (Y.Z.); d.kamato@uq.edu.au (D.K.); 2 Department of Pharmacy, Xinhua College of Sun Yat-sen University, Tianhe District, Guangzhou 510520, China; 3 Sunshine Coast Health Institute, University of the Sunshine Coast, Birtinya, QLD 4575, Australia; 4 Department of Endocrinology, First Affiliated Hospital, Division of Life Sciences and Medicine, University of Science and Technology of China, Hefei 230037, China; suowen.xu@gmail.com

**Keywords:** transforming growth factor receptor, Smad2, inflammation, monocyte chemoattractant protein-1, atherosclerosis, vascular smooth muscle cells

## Abstract

Curcumin is a natural compound that has been widely used as a food additive and medicine in Asian countries. Over several decades, diverse biological effects of curcumin have been elucidated, such as anti-inflammatory and anti-oxidative activities. Monocyte chemoattractant protein-1 (MCP-1) is a key inflammatory marker during the development of atherosclerosis, and curcumin blocks MCP-1 expression stimulated by various ligands. Hence, we studied the action of curcumin on lysophosphatidic acid (LPA) mediated MCP-1 expression and explored the specific underlying mechanisms. In human vascular smooth muscle cells, LPA induces Rho-associated protein kinase (ROCK) dependent transforming growth factor receptor (TGFBR1) transactivation, leading to glycosaminoglycan chain elongation. We found that LPA also signals via the TGFBR1 transactivation pathway to regulate MCP-1 expression. Curcumin blocks LPA mediated TGFBR1 transactivation and subsequent MCP-1 expression by blocking the ROCK signalling. In the vasculature, ROCK signalling regulates smooth muscle cell contraction, inflammatory cell recruitment, endothelial dysfunction and vascular remodelling. Therefore, curcumin as a ROCK signalling inhibitor has the potential to prevent atherogenesis via multiple ways.

## 1. Introduction

Lysophosphatidic acid (LPA) is a pleiotropic agonist frequently associated with atherosclerotic cardiovascular disease [1]. LPA signals via six G protein-coupled receptors (GPCRs) (LPAR1-LPAR6) [2]. We have identified that in human vascular smooth muscle cells (VSMCs), LPA signals via LPAR1 and LPAR5 to stimulate genes that modify the glycosaminoglycan (GAG) chains on proteoglycans [3]. Modified GAG chains bind and trap low density lipoprotein (LDL) inside the intima and thus initiate the development of atherosclerosis [4,5,6]. The trapped LDL undergoes oxidization, resulting in a range of lipid metabolites, including LPA. These metabolites induce inflammatory chemokines that attract leukocytes, such as monocytes, into the intima. Monocytes engulf the oxidized LDL and convert them into macrophages, which advance the development of the atherosclerotic lesions [7]. 

Monocyte chemoattractant protein-1 (MCP-1/CCL2) is a member of the C-C chemokine family, and the receptor(s) for MCP-1 is CCR2, which mediates monocyte chemotaxis via regulation of calcium mobilisation and inhibition of adenylyl cyclase [8]. MCP-1 is considered as a marker and therapeutic target of atherosclerosis [9,10]. The mRNA expression of the MCP-1 is upregulated in human atherosclerotic plaques [11]. In animal models of atherosclerosis, the disruption of MCP-1 signalling reduces lipid deposition and macrophage infiltration within the aortic walls [12] and decreases the atherosclerotic lesion size [13]. In LDL receptor-deficient mice models, the administration of LPAR1/3 antagonist Ki16425 reduces the development of atherosclerotic plaques along with the reduction of MCP-1 [14], suggesting the therapeutic potential of targeting the LPA-MCP-1 signalling axis. LPA is able to stimulate MCP-1 expression in human VSMCs, although the underlying mechanisms are not completely clear [15,16]. LPA via LPAR5 transactivates transforming growth factor-β (TGF-β) receptor (TGFBR1) and leads to GAG chain synthesizing gene expression [3]. Furthermore, TGF-β signalling is associated with vascular inflammation and atherosclerosis [17]. Therefore, we studied the contribution of TGFBR1 transactivation pathway to LPA mediated MCP-1 expression.

Curcumin has diverse roles in the vascular system and has been the subject of clinical investigations for the treatment of cardiovascular disease [18]. Anti-inflammatory effects of curcumin date back to 1970s, where curcumin reduced acute and chronic inflammation in rats, mice and cats [19]. In addition, a meta-analysis of 10 randomized controlled trials showed that a combination of curcuminoid and piperine reduced inflammatory markers, such as C-reactive protein [20]. Curcumin inhibits oxidised LDL [21] and lipopolysaccharide (LPS) [22], angiotensin II [23] and aldosterone [24] induced inflammation in rat VSMCs. Our aim was to study the effect of curcumin on LPA mediated MCP-1 expression in human VSMCs and to define the underlying mechanisms.

## 2. Results

### 2.1. LPA Stimulates MCP-1 via TGFBR1 Transactivation Pathway in VSMCs

**Figure 1:**
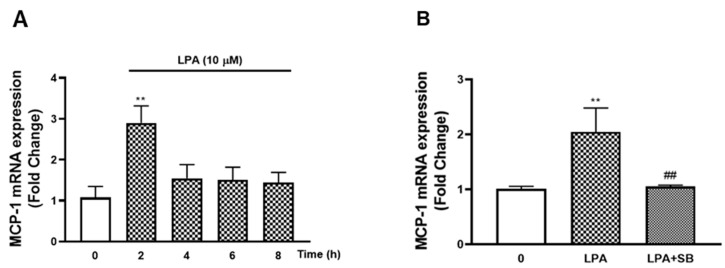
LPA stimulation of MCP-1 and the role of the TGFBR1 transactivation pathway. (**A**) VSMCs were treated with LPA (10 µM) over an 8 h time course. (**B**) VSMCs were pre-incubated with 3 μM SB 431,542 for 30 min before treatment with LPA (10 µM) for 2 h. Total RNA was harvested and assessed by qRT-PCR analysis. 18S was used as a house keeping gene. Results are expressed as mean ± SEM from three independent experiments. Statistical significance was determined by one-way ANOVA, followed by least significant difference post-hoc analysis. ** *p* < 0.01 versus basal; ## *p* < 0.01 versus LPA only treated samples.

In human VSMCs, we found LPA signals via TGFBR1 to stimulate genes associated with the initiation of atherosclerosis [3]. MCP-1 is a critical chemokine in the development of atherosclerosis [10], and LPA is able to stimulate MCP-1 expression [15,16]. We studied the effect of LPA on MCP-1 mRNA expression and the involvement of the TGFBR1 transactivation pathway. VSMCs treated with LPA (10 µM, 2 h) increased the mRNA expression of MCP-1 to 2.9-fold (*p* < 0.01) (Figure 1A). A slight decrease to 1.5-fold was observed at 4 h, and then the level of mRNA remained over 8 h treatment, demonstrating that LPA stimulates MCP-1 mRNA expression in human VSMCs. To study the involvement of TGFBR1 transactivation signalling in LPA mediated MCP-1 expression, a specific TGFBR1 kinase inhibitor (SB431542) was utilised (Figure 1B). VSMCs were treated with LPA (10 µM, 2 h) in the presence or absence of SB431542. The presence of SB431542 completely blocked LPA mediated mRNA increase of MCP-1, demonstrating that LPA acts via the TGFBR1 transactivation pathway to stimulate MCP-1 expression.

### 2.2. The Effect of Curcumin on VSMC Viability

**Figure 2:**
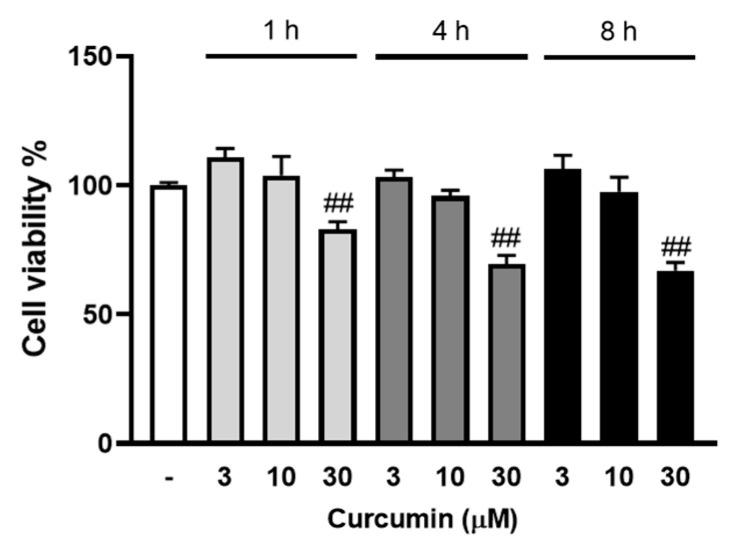
The effect of curcumin on VSMC viability. VSMCs were cultured in 96-well plates and treated with 3, 10 and 30 μM curcumin (in 90 μL media) for 1, 4 and 8 h followed by the AlamarBlue viability assay. The results are representative of three independent experiments (presented as mean ± SEM). Statistical significance was determined by one-way ANOVA, followed by least significant difference post-hoc analysis. ## *p* < 0.01 versus untreated control.

Curcumin blocks MCP-1 expression in vitro and in vivo [25]; therefore, we sought to characterise the role and mechanisms of curcumin in LPA mediated MCP-1 expression. To exclude any involvement of cell toxicity in the cellular actions of curcumin, we assessed the cell viability effect of curcumin at varied conditions. VSMCs were treated with curcumin (3, 10, 30 μM) for 1 to 8 h, and cell viability was assessed using AlamarBlue (Figure 2). Curcumin at 3 to 10 μM had no effect on VSMC viability, whereas 30 μM curcumin showed a 17% reduction in cell viability at 1 h and a 33% reduction at the 8 h time point. For subsequent experiments, we used the maximum tolerated concentration of curcumin (10 μM) in VSMCs.

### 2.3. Curcumin Inhibits LPA Mediated MCP-1 Expression

**Figure 3:**
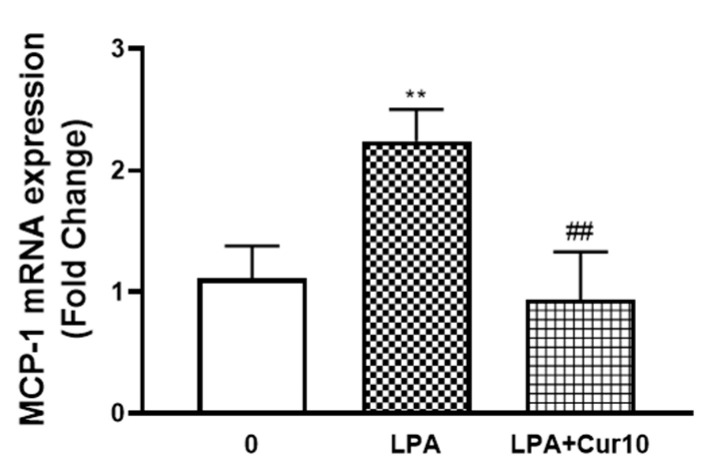
The effect of curcumin on LPA stimulated MCP-1 expression. VSMCs were pre-incubated with 10 μM curcumin for 30 min before treatment with LPA (10 µM) for 2 h. Total RNA was harvested and assessed by qRT-PCR analysis. *18S* was used as a house keeping gene. Results are expressed as mean ± SEM from three independent experiments. Statistical significance was determined by one-way ANOVA, followed by least significant difference post-hoc analysis. ** *p* < 0.01 versus basal; ## *p* < 0.01 versus LPA treated sample.

We then investigated whether curcumin inhibits LPA mediated MCP-1 in VSMCs. LPA (10 µM, 2 h) treated VSMCs increased MCP-1 mRNA expression to 2.2-fold (*p* < 0.01), and the presence of curcumin completely blocked this response (Figure 3), demonstrating that curcumin is an inhibitor of MCP-1 expression in LPA stimulated human VSMCs.

### 2.4. Curcumin Inhibits LPA Mediated Smad2 Phosphorylation

**Figure 4:**
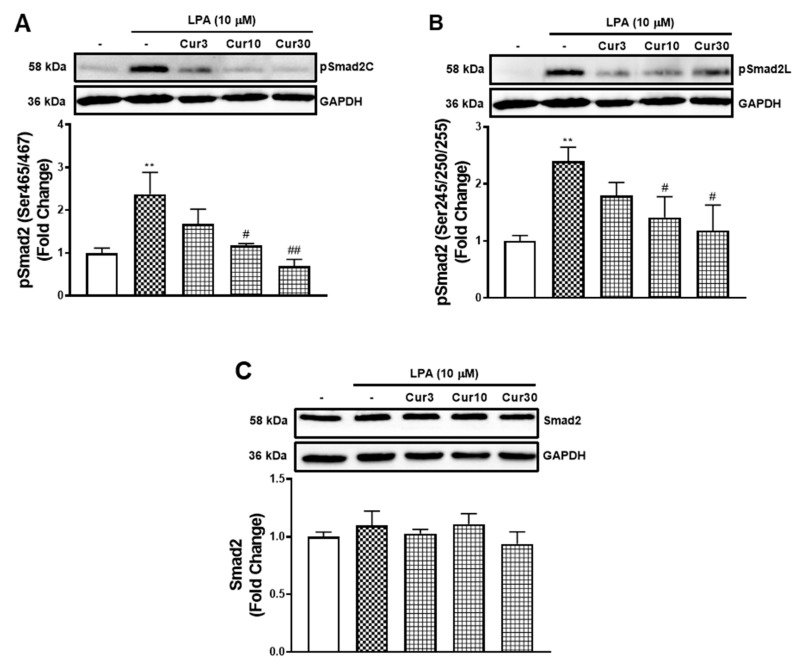
The effect of curcumin on LPA stimulated Smad2 signalling. VSMCs were pre-incubated 3, 10 and 30 μM curcumin for 30 min before treatment of LPA (10 μM) for 30 min. Blots were probed with primary antibodies specific to (**A**) phospho Smad2 (Ser465/467) (1:1000), (**B**) phospho Smad2 (Ser245/250/255) (1:1000), (**C**) Smad2 (1:1000) and secondary HRP conjugated rabbit IgG antibody (1:2000). Blots are representative of three independent experiments. Figure A and B share the same blot of GAPDH. Histogram represents band density expressed as fold per basal (presented as mean ± SEM) after normalised to GAPDH. Statistical significance was determined by one-way ANOVA, followed by least significant difference post-hoc analysis. ** *p* < 0.01 versus basal; # *p* < 0.05 and ## *p* < 0.01 versus LPA only treated samples.

LPA acted via the TGFBR1 transactivation pathway to stimulate the mRNA expression of MCP-1 that was inhibited by curcumin. Here, we investigated whether curcumin inhibits the TGFBR1 transactivation pathway. TGFBR1 activation directly causes the phosphorylation of Smad2 on the carboxyl terminal (pSmad2C) [26] and indirectly leads to the phosphorylation of the linker region (pSmad2L) [27]. To characterise the actions of curcumin on LPA transactivation dependent signalling, we measured its effect on LPA mediated pSmad2C (Figure 4A) and pSmad2L (Figure 4B). LPA treatment of VSMCs activated TGFBR1, leading to an increase of pSmad2C levels by 2.4-fold (*p* < 0.01). The presence of curcumin dose-dependently inhibited LPA mediated pSmad2C, with 87% (*p* < 0.05) inhibition observed by 10 μM curcumin. Similarly, LPA treated VSMCs stimulated pSmad2L to 2.4-fold (*p* < 0.01). The presence of curcumin dose-dependently inhibited LPA mediated pSmad2L, with a 70% (*p* < 0.05) inhibition observed by 10 μM curcumin. To study whether the reduction of pSmad2 is due to increased protein degradation, we also analysed the level of total Smad2 in VSMCs after curcumin treatment (Figure 4C). The treatment of LPA and curcumin showed no effect on the level of Smad2, suggesting curcumin blocks the TGFBR1 transactivation signalling pathway that regulates the phosphorylation of Smad2, not signalling pathways that regulate the level of Smad2 within the cells.

### 2.5. Curcumin Shows no Effect on LPA Mediated MAPK Signalling at the Tolerated Concentrations

**Figure 5:**
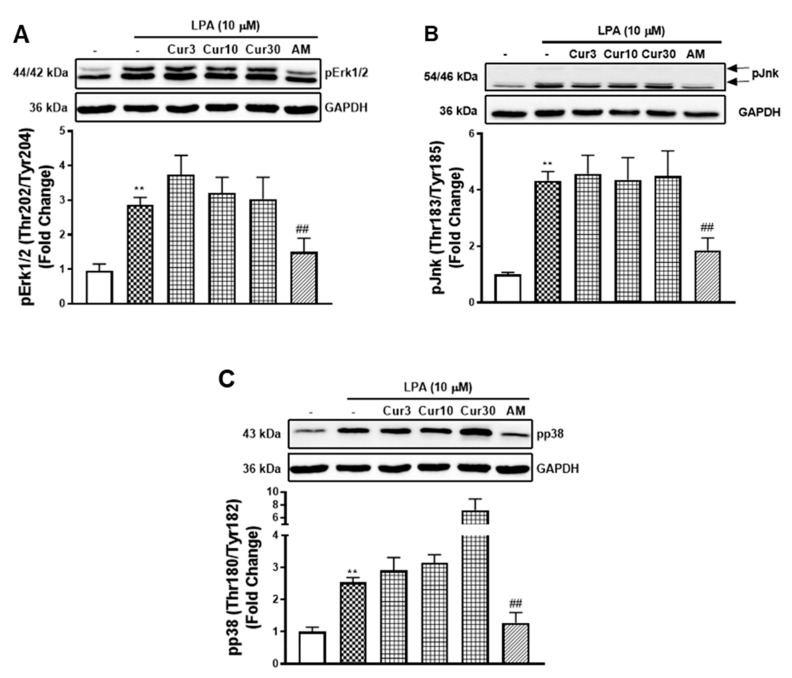
The effect of curcumin on LPA stimulated MAPK signalling. VSMCs were pre-incubated with 3, 10 and 30 μM curcumin for 30 min before the treatment of LPA (10 μM) for 30 min. Blots were probed with primary antibodies specific to (**A**) phospho-Erk (Thr202/Tyr204) (1:4000), (**B**) phospho-Jnk (Thr183/Tyr185) (1:1000), (**C**) phospho-p38 (Thr180/Tyr182) (1:2000) and secondary HRP conjugated rabbit IgG antibody (1:2000). Blots are representative of three-independent experiments. Figures A and C share the same blot of GAPDH. Histogram represents band density expressed as fold per basal (presented as mean ± SEM) after normalised to GAPDH. Statistical significance was determined by one-way ANOVA, followed by least significant difference post-hoc analysis. ** *p* < 0.01 versus basal; ## *p* < 0.01 versus LPA only treated samples.

The phosphorylation of the Smad2L is activated by multiple serine and threonine kinases, including mitogen-activated protein kinases (MAPKs) [28,29,30]. To estimate the roles of MAPKs in curcumin inhibition of LPA mediated Smad2L phosphorylation and MCP-1 expression, we studied the effect of curcumin on LPA mediated MAPKs (Figure 5A–C). LPA treated VSMCs showed a 2.9-fold (*p* < 0.01) increase in pErk1/2 that was unaffected when treated with different concentrations of curcumin. LPAR1 inhibitor, AM095, partially decreased LPA stimulated pErk1/2 (Figure 5A). Similarly, LPA treated VSMCs stimulated pJnk to 4.3-fold (*p* < 0.01) that was unaffected by curcumin but partially inhibited by AM095 (Figure 5B). LPA treated VSMCs stimulated pp38 to 2.5-fold (*p* < 0.01) that was unaffected by curcumin at 3 and 10 μM. However, a further stimulation was observed at 30 μM, which might be explained by the cell toxicity of curcumin at this concentration. LPAR1 inhibitor AM095 attenuated LPA stimulated pp38 as expected (Figure 5C). The data shows that curcumin inhibition of LPA mediated Smad2L signalling and MCP-1 expression is not occurring via the MAPK signalling pathways. We proceeded to investigate other possible mechanisms.

### 2.6. Curcumin Inhibits LPA Mediated MCP-1 Expression by Blocking ROCK Signalling

**Figure 6:**
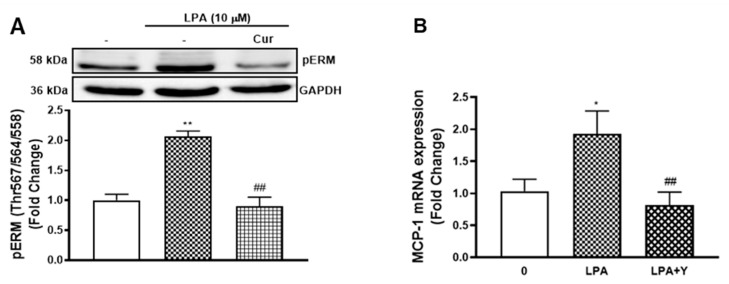
Curcumin inhibits LPA initiated MCP-1 expression via blocking ROCK signalling. (**A**) VSMCs were pre-incubated with 10 μM curcumin for 30 min before treatment of LPA (10 μM) for 15 min. Blots were probed with primary antibodies specific to phospho-Ezrin (Thr567)/Radixin (Thr567)/Moesin (Thr558) (1:2000) and secondary HRP conjugated rabbit IgG antibody (1:2000). Blots are representative of three independent experiments. (**B**) VSMCs were pre-incubated with 10 μM Y27632 for 30 min before treatment with LPA (10 µM) for 2 h. Total RNA was harvested and assessed by qRT-PCR. *18S* was used as a house keeping gene. Results are expressed as mean ± SEM from three independent experiments. Statistical significance was determined by one-way ANOVA, followed by least significant difference post-hoc analysis. * *p* < 0.05 and ** *p* < 0.01 versus basal; ## *p* < 0.01 versus LPA only treated sample.

We have identified that LPA mediated TGFBR1 transactivation is ROCK signalling dependent as the use of ROCK inhibitor (Y27632) blocks LPA stimulated pSmad2C [3]. Curcumin inhibits ROCK signalling in different experimental models [31,32]. To investigate if curcumin blocks the TGFBR1 transactivation pathway via the ROCK signalling, we first characterised the effect of curcumin on LPA phosphorylated ezrin/radixin/moesin (pERM), an immediate downstream of ROCK signalling. LPA treated VSMCs showed a 2.0-fold increase of pERM (*p* < 0.01) (Figure 6A) that was completely blocked by curcumin, suggesting that curcumin blocks the TGFBR1 transactivation pathway via suppressing the ROCK signalling.

To investigate if curcumin was acting via inhibition of ROCK dependent signalling to regulate LPA mediated MCP-1 expression, we measured the mRNA level of MCP-1 in the presence and absence of the ROCK inhibitor, Y27632. LPA treatment of VSMCs stimulated MCP-1 expression to 1.9-fold (*p* < 0.01), and Y27632 completely blocked this response (Figure 6B). This data demonstrates that curcumin acts via inhibiting ROCK signalling dependent pathway to block LPA mediated MCP-1 expression.

## 3. Discussion

**Figure 7:**
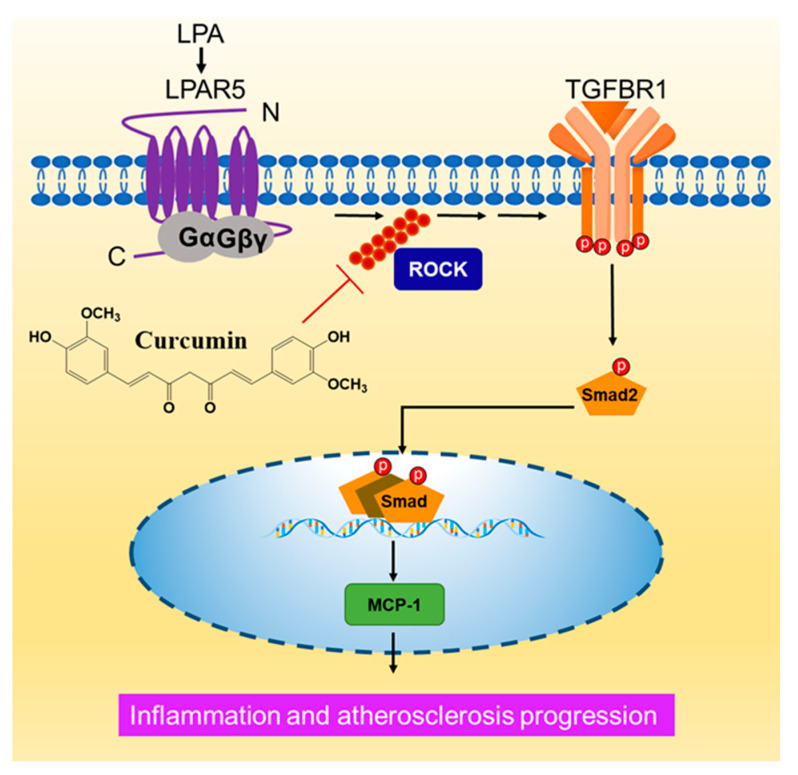
A schematic showing the mechanism of curcumin inhibiting of LPA mediated MCP-1 expression. In VSMCs, LPA via LPAR5 transactivates the TGFBR1 in a ROCK dependent manner. LPA mediated MCP-1 expression is regulated by the TGFBR1 transactivation pathway. Curcumin blocks LPA mediated TGFBR1 transactivation and MCP-1 expression via inhibiting ROCK signalling.

Atherosclerosis is a pathogenic process featured with augmented inflammatory responses. In this paper, we studied the effect of LPA on inflammatory marker MCP-1 expression and the effect of curcumin in human VSMCs. We have previously shown that LPA via LPAR5 initiates ROCK signalling dependent TGFBR1 transactivation to regulate GAG chain synthesizing gene expression [3]. Here, we showed that LPA acts via the TGFBR1 transactivation pathway to stimulate the gene expression of MCP-1 in human VSMCs. We identified that curcumin blocks LPA mediated MCP-1 expression via suppressing the ROCK dependent TGFBR1 transactivation pathway (Figure 7).

MCP-1 promotes the development of atherosclerosis mainly by recruiting monocytes or macrophages into the vascular wall [10]. In human umbilical cord vein endothelial cells (HUVECs), LPA stimulates the mRNA and protein expression of MCP-1 [33]. We found that LPA also stimulates the expression of MCP-1 in VSMCs, which is consistent with previous observations [15]. However, the underlying mechanisms have yet to be completely revealed. In the aortic wall of an aged rat, MCP-1 is increased and co-localized with TGF-β. In addition, the treatment of TGF-β dose-dependently increases MCP-1 expression in the young rat VSMCs [34]. In another rat VSMC model, TGF-β stimulates MCP-1 expression through the Smad3 dependent pathway [35]. TGF-β treatment or the overexpression of Smad3 significantly increases MCP-1 expression that was inhibited by SB431542. Similarly, in HUVECs, TGF-β via the Smad3 dependent pathways stimulates MCP-1 expression [36], and mechanistic studies demonstrate the binding of Smad3 and Smad4 to the promoter region of MCP-1. These lines of data demonstrate that TGFBR1-Smad signalling is a relevant regulator of MCP-1 expression. We previously studied the role of LPA transactivation dependent signalling in VSMCs, and our studies demonstrate that LPA via its LPAR5 transactivates the TGFBR1 [3]. Here, LPA mediated MCP-1 expression was completely blocked by TGFBR1 kinases inhibitor, suggesting the involvement of the TGFBR1 transactivation pathway in this response.

Curcumin is well-known for its anti-inflammatory pharmacological effects [18]. Curcumin completely blocked LPA mediated MCP-1 expression in human VSMCs. Curcumin also blocked oxidised LDL [21] and LPS stimulated MCP-1 expression in rat VSMCs [22]. These observations support that curcumin is a potent inhibitor of MCP-1 expression in different cell lines induced by varied ligands [25]. MCP-1 is a trigger of atherosclerosis through inducing leukocyte infiltrate into the sub-endothelial; therefore, curcumin could be a potential drug for the prevention of atherosclerosis at least via attenuating the level of MCP-1. The underlying molecular mechanisms will be worth exploring.

We studied the effect of curcumin on the LPA mediated TGFBR1 transactivation pathway. Curcumin dose-dependently inhibits LPA stimulation of pSmad2C and pSmad2L, suggesting the capability of curcumin to inhibit the TGFBR1 transactivation pathway. MAPKs are involved in the phosphorylation of Smad2L in human VSMCs [28,29,37]. In addition, the MAPK signalling dependent pathways are also associated with inflammatory responses [38]. In rat VSMCs, curcumin reduces oxidised LDL mediated MCP-1 expression through down-regulating p38 MAPK [21]. However, we did not observe any inhibitory effect from curcumin at the tolerated concentrations by measuring LPA phosphorylated Erk1/2, Jnk and p38. This data show that curcumin exerts its biological function via TGFBR1 at the tolerated concentrations independent of MAPKs. 

We identified that thrombin and LPA mediate TGFBR1 transactivation via the ROCK-integrin pathway [3,26]. The activation of ROCK signalling leads to cytoskeletal rearrangement, which activates cell surface integrins. The activated integrins lead to confirmational changes of the TGF-β complex, allowing for the ligand to interact with its receptor and activate downstream Smad signalling [26]. Therefore, agents that inhibit the biochemical mechanisms involved in TGFBR1 transactivation could be explored for therapeutic potential to ameliorate LPA mediated TGFBR1 complications. Curcumin has the potential to inhibit ROCK1 [31,39]. In cultured DRG neurons, curcumin reduces the phosphorylation of the LIMK1 kinase, a substrate of ROCK, suggesting the potential role of curcumin to inhibit ROCK signalling dependent pathways [40]. Specially, curcumin reduces LPA activated RhoA and ROCK in MCF7 breast cancer cells [32]. These lines of evidence demonstrate that curcumin has the potential to inhibit ROCK signalling and cytoskeletal rearrangement, and therefore inhibition of TGFBR1 transactivation. Our data show that curcumin dose inhibits the phosphorylation of ezrin, radixin and moesin (ERM), a downstream target of ROCK. Moreover, LPA mediated pSmad2C and pSmad2L were also blocked by curcumin, indicating curcumin blocks LPA mediated TGFBR1 transactivation via inhibiting ROCK signalling.

Curcumin can also block the TGFBR1 downstream signalling [41], including TGF-β mediated phosphorylation of Smad2 and Smad3 [42]. To further investigate whether curcumin inhibition of MCP-1 expression occurs via inhibiting the transactivation pathway not the TGFBR1 downstream, a ROCK inhibitor (Y27632) was used [43]. The ROCK inhibitor completely blocked LPA stimulated MCP-1, suggesting the regulatory role of the ROCK dependent pathway. In human aortic endothelial cells (HAECs), Y27632 broadly blocks LPA mediated inflammatory markers including MCP-1 [44]. Gene silencing of ROCK2 but not ROCK1 results in an attenuation of MCP-1, indicating that the ROCK2 isoform is required for LPA mediated inflammatory responses in these cells [44]. LPA stimulated MCP-1 in HUVECs is Rho dependent, as exotoxin C3, a specific inhibitor of Rho, inhibited MCP-1 protein expression [33]. In another human aortic SMC, LPA stimulated MCP-1 is Rac-1 dependent as the transfection of negative Rac-1 mutant inhibited this response [16]. Rho is an upstream of ROCK, while Rac-1 regulates the downstream of ROCK and cytoskeleton rearrangement [45,46]. Therefore, these data together with our observation support that LPA mediated MCP-1 expression is ROCK signalling dependent. Together, in VSMC, curcumin blocks LPA mediated MCP-1 via suppressing the ROCK dependent pathways.

## 4. Materials and Methods

### 4.1. Materials

Human aortic VSMCs (ATCC^®^ CRL-1999™) were purchased from In Vitro Technologies Life science (Melbourne, Australia). Ham’s F-12 K (Kaighn’s) medium, foetal bovine serum (FBS), GlutaMAX^TM^-I (100×), antibiotics solution (10,000 U/mL penicillin, 10,000 μg/mL streptomycin), 0.25% trypsin-EDTA and AlamarBlue were purchased from Thermo Fisher Scientific (Melbourne, Australia). LPA (CAS: 22556-62-3), curcumin, AM095, Y27632, SB431542 and endothelial cell growth supplement (ECGS) were purchased from Sigma Aldrich (Sydney, Australia). Antibodies to phospho-Smad2 (Ser465/467) (3108S), phospho-Smad2 (Ser245/250/255) (3104S), Smad2 (3122S), phospho-Erk (Thr202/Tyr204) (4377S), phospho-Jnk (Thr183/Tyr185) (4668S), phospho-p38 (Thr180/Tyr182) (9215S), phospho-Ezrin (Thr567)/Radixin (Thr567)/Moesin (Thr558) (3141S), rabbit immunoglobulin-G (IgG) horseradish peroxidase (HRP)-linked antibody (7074S) and glyceraldehyde-3-phosphate dehydrogenase (GAPDH) (3683S) were purchased from Australian Bioresearch (Perth, Australia). Primers for RRN18S and MCP-1, RNeasy^®^ Mini Kit, QuantiTect^®^ Reverse Transcription Kit and QuantiNova™ SYBR^®^ Green PCR Kit were purchased from Qiagen (Melbourne Australia). 

### 4.2. Cell Culture

Human VSMCs were grown in complete Ham’s F-12K medium (10% FBS and 1% antibiotics, 5% GlutaMAX, 0.3 mg/mL ECGS) at 37 °C with 5% CO_2_. For experiments, cells were seeded into 60 mm dishes or 96-well plates and were grown to confluence, then rendered quiescent using F-12K medium (0.1% FBS and 1% antibiotics) for 48 h. Treatment details are given in the figure legends.

### 4.3. Cell Viability Assay

The cell viability was estimated by AlamarBlue assay. VSMCs were seeded in 96-well plates at 1.0 × 10^4^ cells per well and rendered quiescent the next day for 48 h before curcumin treatment. Cells were treated with curcumin (in 90 μL media) for a desired time. Subsequently, the AlamarBlue reagent (10 μL) was added to each well, and the plates were incubated at 37 °C with 5% CO_2_ for 2 h. Viability was then analysed by detection of absorbance at 570 nm using 600 nm as a reference wavelength.

### 4.4. Western Blotting

Protein expression level was analysed as previously described [3]. Protein lysates were separated on a 10% SDS-PAGE and protein bands were semi-dry transferred onto PVDF membranes. Membranes were blocked by 5% BSA before the incubation of primary antibodies. All primary antibodies were diluted using 5% BSA in TBST buffer and incubated overnight at 4 °C except pErk1/2 antibody and GAPDH, which were incubated for 1 h at room temperature. GAPDH was used as the loading control. Then, membranes were incubated with secondary antibody (HRP-anti-rabbit IgG) at room temperature for 1 h. Blots were imaged using enhanced chemiluminescence detection on the Bio-Rad gel documentation system (BioRad, Sydney Australia), and densitometry analysis was performed using Image Lab 5.2.1 (BioRad, Sydney Australia).

### 4.5. Quantitative Real Time Polymerase Chain Reaction (qRT-PCR)

mRNA expression level of targeted genes was analysed using qRT-PCR as previously described [3]. RNeasy Mini kits were used to isolate and purify the cellular RNA. Quantitect reverse transcriptase kits were used to synthesize first strand cDNA. QuantiNova SYBR green PCR master mix kits were used to perform qRT-PCR on Qiagen Rotor Gene Q. Data was normalised to the ribosomal *18S* housekeeping gene. Relative mRNA expression of genes was quantified using the comparative delta delta Ct method. 

### 4.6. Statistical Analysis

Data were presented as the mean ± standard error of the mean (SEM) of three independent experiments. Data statistical significance was analysed as previously described [3].

## 5. Conclusions

We observed that LPA via TGFBR1 transactivation pathway stimulates MCP-1 expression in human VSMCs. In addition, we identified that curcumin attenuates LPA mediated MCP-1 expression via inhibiting the ROCK signalling and subsequent TGFBR1 transactivation. Findings of our study in addition to the literature suggest that curcumin could be a broad inhibitor of MCP-1 expression. Our identification of ROCK signalling as a target of curcumin might explain the multitude of disease ameliorating actions that have been described for curcumin. The activation of ROCK signalling in various vasculature cells regulates vascular inflammation and remodelling. Therefore, curcumin could be a potential drug to prevent atherosclerosis by suppressing ROCK signalling and diverse subsequent cellular responses.

## Data Availability

The data that support the findings of this study are available from the corresponding author upon reasonable request.

## Sample Availability

Samples used in this paper are commercially available.
